# Factors Affecting Glomerular Filtration Rate, as Measured by Iohexol Disappearance, in Men with or at Risk for HIV Infection

**DOI:** 10.1371/journal.pone.0086311

**Published:** 2014-02-07

**Authors:** Joseph B. Margolick, Lisa P. Jacobson, George J. Schwartz, Alison G. Abraham, Annie T. Darilay, Lawrence A. Kingsley, Mallory D. Witt, Frank J. Palella

**Affiliations:** 1 Department of Molecular Microbiology and Immunology, Johns Hopkins Bloomberg School of Public Health, Baltimore, Maryland, United States of America; 2 Department of Epidemiology, Johns Hopkins Bloomberg School of Public Health, Baltimore, Maryland, United States of America; 3 Department of Pediatrics, University of Rochester Medical Center, Rochester, New York, United States of America; 4 Department of Infectious Diseases and Microbiology and Department of Epidemiology, University of Pittsburgh, Pittsburgh, Pennsylvania, United States of America; 5 Department of Medicine, David Geffen School of Medicine at University of California Los Angeles, Los Angeles, California, United States of America; 6 Los Angeles Biomedical Research Institute at Harbor-UCLA, University of California Los Angeles, Los Angeles, California, United States of America; 7 Department of Medicine, Northwestern University Feinberg School of Medicine, Chicago, Illinois, United States of America; University of São Paulo School of Medicine, Brazil

## Abstract

**Objective:**

Formulae used to estimate glomerular filtration rate (GFR) underestimate higher GFRs and have not been well-studied in HIV-infected (HIV(+)) people; we evaluated the relationships of HIV infection and known or potential risk factors for kidney disease with directly measured GFR and the presence of chronic kidney disease (CKD).

**Design:**

Cross-sectional measurement of iohexol-based GFR (iGFR) in HIV(+) men (n = 455) receiving antiretroviral therapy, and HIV-uninfected (HIV(−)) men (n = 258) in the Multicenter AIDS Cohort Study.

**Methods:**

iGFR was calculated from disappearance of infused iohexol from plasma. Determinants of GFR and the presence of CKD were compared using iGFR and GFR estimated by the CKD-Epi equation (eGFR).

**Results:**

Median iGFR was higher among HIV(+) than HIV(−) men (109 vs. 106 ml/min/1.73 m^2^, respectively, p = .046), and was 7 ml/min higher than median eGFR. Mean iGFR was lower in men who were older, had chronic hepatitis C virus (HCV) infection, or had a history of AIDS. Low iGFR (≤90 ml/min/1.73 m^2^) was associated with these factors and with black race. Other than age, factors associated with low iGFR were not observed with low eGFR. CKD was more common in HIV(+) than HIV(−) men; predictors of CKD were similar using iGFR and eGFR.

**Conclusions:**

iGFR was higher than eGFR in this population of HIV-infected and -uninfected men who have sex with men. Presence of CKD was predicted equally well by iGFR and eGFR, but associations of chronic HCV infection and history of clinically-defined AIDS with mildly decreased GFR were seen only with iGFR.

## Introduction

As HIV-infected (HIV(+)) persons live longer through use of highly active antiretroviral therapy (HAART) [1], [2], kidney disease has emerged as a significant cause of morbidity and mortality in this population, often in association with other chronic diseases that affect kidneys such as diabetes mellitus and hypertension [3], [4]. In addition, some antiretroviral drugs can reduce kidney function (both glomerular and tubular) [5]–[10]. Use of serum creatinine-based glomerular filtration rate (GFR)-estimating equations to stage kidney disease, guide drug therapy, and evaluate medication-related nephrotoxicity is standard medical practice. However, use of these equations has limitations in HIV(+) persons. First, HIV-related conditions such as wasting, sarcopenia, and reductions in lean body mass can reduce serum creatinine levels, leading to GFR overestimation. Second, many of these equations, notably Cockroft-Gault [11] and Modification of Diet in Renal Disease (MDRD) [12], two of the most commonly used, were derived from persons with renal impairment (GFR ≤90 ml/min/1.73 m^2^), have not been validated among persons with normal kidney function, and tend to underestimate higher GFRs. The CKD-Epi GFR-estimating equation, and other newer equations [13], are more accurate among persons with near-normal kidney function but still tend to underestimate GFR [14], although GFR estimates using this equation correlated well with measured GFR in two small [15], [16] and three larger [17]–[19] studies of HIV(+) persons. Third, estimates of GFR (eGFR) depend heavily on the method of creatinine measurement (enzymatic vs. Jaffe), and these have only recently been referenced to isotope dilution mass spectrometry standards [20]. No studies to date have compared predictors of reduced GFR and chronic kidney disease (CKD) using the CKD-Epi equation with predictors obtained using directly measured GFR.

A more accurate measurement of GFR may be needed to better define the impact upon kidney function of antiretroviral therapy, chronic co-morbid diseases, and aging among HIV(+) adults, especially in the early stages of renal disease. To this end, we directly measured GFR in a large well-characterized cohort of men who were either HIV(+) and receiving HAART, or HIV(−) but of similar lifestyles. Direct measurement of GFR was based on the disappearance from plasma of the contrast dye iohexol, which after intravenous infusion is excreted almost exclusively through glomerular filtration [21], [22]. This method has been validated across all levels of kidney function in human cohort studies [21]–[24], but in only two studies of HIV(+) individuals [13], [19]. We then investigated determinants of lower GFR, both directly measured and estimated, among HIV(+) and HIV(−) men followed in the Multicenter AIDS Cohort Study (MACS).

## Methods

### Study Population

Participants in the Multicenter AIDS Cohort Study (MACS), an ongoing observational study of HIV infection in men who have sex with men, were recruited for iGFR testing. Overall, 6972 HIV(+) and HIV(−) men were enrolled from 1984 to 2003 in Baltimore/Washington D.C., Chicago, Los Angeles and Pittsburgh [25]–[27]. MACS semiannual study visits include standardized questionnaires, physical examinations and blood and urine capture for laboratory analyses and storage. For this study, HIV(−) men and HIV(+) men receiving HAART were selected randomly in a 1∶2 ratio. Participants who received renal replacement therapy, had been diagnosed with cancer in the preceding 3 years, or were allergic to contrast material were excluded. All MACS participants with hepatitis C virus (HCV) infection (defined by presence of circulating anti-HCV antibody confirmed by detection of HCV RNA as described [28]) were eligible to participate. HCV antibody-positive participants with undetectable plasma HCV RNA for >3 years were considered HCV-uninfected. For the present study, 101 men had confirmed HCV infection and were included in the study, including two who had negative HCV RNA tests at the GFR study visit but had had detectable HCV RNA within three years of this visit. Chronic hepatitis B infection was assessed as described [29]. HIV seropositivity was defined by a positive ELISA confirmed by Western blot. Plasma HIV RNA levels were measured by the Roche Amplicor assay (Hoffman-LaRoche, Nutley, NJ) sensitive to 50 copies/ml, CD4 T-lymphocyte counts by standardized flow cytometry [30], and serum creatinine concentrations by high performance liquid chromatography (HPLC) [31].

### Measurement of Iohexol-based GFR (iGFR)

As described [32]–[34], iohexol concentrations were measured by HPLC (University of Rochester Medical Center GFR Laboratory) in blood specimens drawn 10, 30, 120, and 240 minutes after intravenous infusion of 5 ml (∼3200 mg) of iohexol [32], [33]. GFR was calculated using a two-compartment model describing the fast and slow components of the decay of plasma iohexol concentration over time, as described [32], [33], [35], and normalized to calculated body surface area (BSA) [36]. Occasionally (7% of cases), when the intercept or slope of the fast curve could not be calculated, iGFR was calculated from the decay of the slow compartment (120- and 240-minute iohexol concentrations), as described [34]. In two cases, the iohexol dose infused was unknown, and was taken as 3200 mg, the nominal dose. The median dose received by the other participants was 3175 mg (interquartile range 3127–3224 mg).

### Covariates and Statistical Analysis

Data from the semi-annual MACS study visit closest to the iGFR measurement were used to define current behaviors and physical attributes. Hypertension, diabetes and dyslipidemia were considered present if confirmed at ≥2 visits before the iGFR measurement, according to the following definitions: Hypertension was defined as a systolic blood pressure ≥140 mm Hg or a diastolic blood pressure ≥90 mm Hg or receipt of antihypertensive medication with a self-reported history of hypertension. Similarly, diabetes mellitus was defined as a fasting glucose ≥126 mg/dl, or receipt of glucose-lowering medication with a history of a diagnosis of diabetes. Dyslipidemia was defined as the presence of any of the following fasting measurements: total serum cholesterol ≥200 mg/dl, LDL ≥130 mg/dl, HDL<40 mg/dl, triglycerides ≥150 mg/dl, or receipt of lipid-lowering medication with a history of dyslipidemia. Proteinuria, measured using spot urine assessment of protein/creatinine ratio (Quest Diagnostics) at the study visit closest to the iGFR measurement (mean interval = 0.2 yr, range = 0–2.8 yr, but <1 yr for all but 4 men), was considered present when the urine protein/creatinine ratio was ≥0.2 gm protein/gm creatinine.

AIDS-defining illnesses were defined using the 1993 CDC case definition [37] except for cases identified only by CD4 T-cell counts <200/mm^3^. HAART use was defined using the DHHS/Kaiser Panel guidelines [38]. The date of HAART initiation was defined as halfway between the study visits surrounding the self-reported onset of HAART use. HAART regimens were categorized exclusively according to use of: 1) protease inhibitor (PI); 2) no PI, but non-nucleoside reverse transcriptase inhibitor (NNRTI); and 3) only nucleoside reverse transcriptase inhibitors (NRTIs). We examined use of any of these medications, and of tenofovir disoproxil fumarate (TDF), as predictors of GFR, as well as duration of medication use and current versus past use.

Distributions of characteristics between populations were compared using the Pearson χ^2^-test for categorical variables and the Kruskal-Wallis test (or Mood’s Median test when variances were unequal) for continuous variables. When categorical data were sparse, Fisher’s exact test was performed. To examine associations with GFR ≤90 ml/min/1.73 m^2^ [39] and with chronic kidney disease (CKD, defined as GFR <60 ml/min/1.73 m^2^ and/or proteinuria [39]), multivariate logistic regression models were constructed using risk factors from a priori hypotheses and from univariate analyses; these included age, self-reported race, HCV infection, HIV infection with and without prior AIDS, diabetes mellitus, and hypertension. Analyses limited to HIV(+) men included tenofovir use, duration of HAART use, CD4 T-cell counts, plasma HIV RNA levels, and history of AIDS. Wald tests were used to determine statistical significance and Hosmer-Lemeshow tests were used to assess the fit of the model. Quadratic terms for quantitative predictors were fitted initially in the model to check for non-linearity. Estimated GFR (eGFR) was derived using the CKD-EPI formula [14]. The direction of misclassification using eGFR was assessed by McNemar’s test.

Relationships between GFR analyzed as a continuous variable and exposures of interest were explored using linear regression. Effects of risk factors on mean GFR were estimated by standard least squares methods (or the maximum likelihood method when there was evidence of unequal variances) [40]. The homogeneity of variances assumption was assessed through Levene’s test and residual plots, while linearity and normality assumptions were checked visually using residual plots.

### Ethics Statement

This study was approved by institutional review boards at all participating sites, as follows: the Northwestern University Institutional Review Board, the University of Pittsburgh Institutional Review Board, the Johns Hopkins Bloomberg School of Public Health Institutional Review Board, and the John F. Wolf, Human Subject Committee at the Los Angeles Biomedical Research Institute at Harbor-UCLA. Written informed consent was provided by all study participants.

## Results

From August 2008 to December 2010, 741 men underwent an iohexol study; 715 (96%) had satisfactory iGFR determinations, including 662 (89%) by 4-point determination and 53 (7%) by 2-point determination. The remaining 26 (4%) studies were invalid due to anomalous increases in plasma iohexol concentrations post-infusion (n = 13), missing data (n = 3), infusions with incorrect amounts of iohexol (n = 4), blood samples drawn too early (n = 5), or unknown reasons (n = 1). The proportion of invalid iGFR determinations was similar to that seen in the CKiD study [32] (J. Jerry, personal communication). Two additional HIV(+) men were excluded from analysis because of missing values for CD4 T-cell counts and plasma HIV RNA levels. The 713 men studied were also similar to MACS participants not included in the study, but were more likely to be black and HCV-infected (by design), and had slightly lower serum creatinine values (medians = 0.88 vs. 0.97 mg/dl, respectively; p<0.01).

Among the 713 men studied, HIV(+) men were younger, weighed less, were more likely to have had dyslipidemia and proteinuria, and had higher mean eGFR than HIV(−) men (**Table 1** **)**. Most HIV(+) men had no history of clinically-defined AIDS and had undetectable plasma HIV RNA levels **(** **Table 1** **)**. The correlation between iGFR and eGFR was moderate (r = 0.68 for CKD-Epi and 0.64 for MDRD) and was not affected by HIV serostatus.

**Table 1:** Characteristics of the study sample at the time of iohexol-based glomerular filtration rate (iGFR) determination.

Characteristic		HIV(−)			HIV(+)		Overall
		(N = 258)			(N = 455)		(N = 713)
		Median (IQR) or %					
Black (%)		33			36		35
Age, yr#		54 (48–61)			51 (46–57)		52 (47–58)
Height, m		1.76 (1.71–1.82)			1.76 (1.71–1.80)		1.76 (1.71–1.81)
Weight, kg#		83.4 (74.7–93.6)			79.0 (71.2–88.9)		80.8 (72.4–91.0)
Body-Mass Index, kg/m^2^ #		26.8 (24.2–29.9)			25.8 (23.5–28.5)		26.3 (23.8–29.0)
Body surface area, m^2^ #		2.04 (1.91–2.19)			1.98 (1.86–2.11)		2.01 (1.87–2.14)
Serum creatinine,^ mg/dl^		0.88 (0.78–1.01)			0.87 (0.75–1.02)		0.88 (0.76–1.01)
Proteinuria# (%)		4.7			19.3		14.0
HCV-infected (%)		12			15		14
History of diabetes mellitus (%)		14			18		17
History of hypertension (%)		70			66		67
History of dyslipidemia# (%)		88			96		93
GFR (MDRD) (ml/min/1.73 m^2^)#		95 (82–110)			99 (85–117)		97 (83–115)
GFR (CKD-Epi) (ml/min/1.73 m^2^)#		98 (86–109)			102 (91–113)		101 (88–111)
GFR (iohexol) (ml/min/1.73 m^2^)		106 (96–119)			109 (92–125)		107 (94–123)
History of AIDS (%)					15		
HIV RNA <50 copies/mL (%)					80		
CD4 lymphocyte count (cells/uL)					536 (384–737)		
Tenofovir use (%)	Never			22			
	Former			14			
	Current			64			

HCV = hepatitis C virus.

# P-value <0.05 (χ^2^-test or Kruskal-Wallis or Median test for comparison between HIV(−) and HIV(+)).

### HIV, age, and GFR

**Figure 1:**
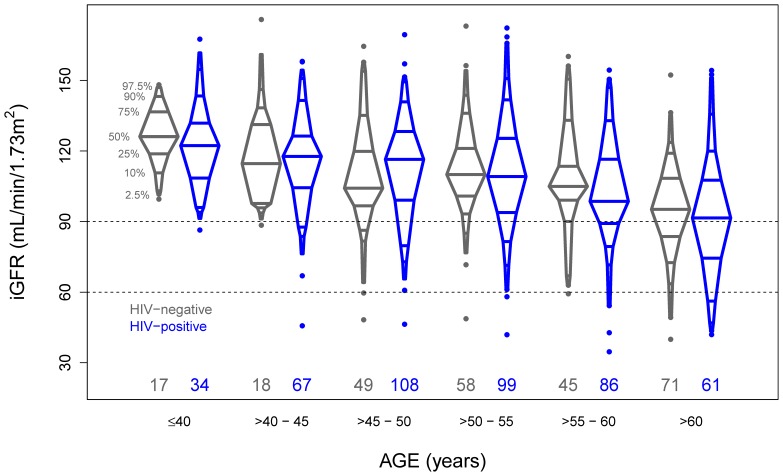
Distribution of iohexol-based GFR (iGFR) by age and HIV serostatus. Boxplots indicate distribution of iGFR values for each category of age (in 5 year increments) and HIV serostatus; gray figures for HIV-uninfected and black figures for HIV-infected. Percentiles that are presented are the 2.5%, 5%, 10%, 25%, 50%, 75%, 90%, 95% and 97.5%. Horizontal dashed lines indicate 90 and 60 ml/min/1.73 m^2^. The percentages of the data for each box that are below 90 ml/min/1.73 m^2^ are given. The numbers (N) of observations contributing to each box are provided at the bottom of the graph.

The overall study population had a median iGFR of 107.4 ml/min/1.73 m^2^. HIV(+) men had slightly higher iGFRs than HIV(−) men, and for both groups the median iGFR was 7–10% higher than the median eGFR (**Table 1**). As shown in **Figure 1**, iGFR declined with age in both HIV(+) and HIV(−) men, and medians by age were similar for both groups, except among men aged 45–50 years. However, across all ages, more HIV(+) men had iGFRs ≤90 ml/min/1.73 m^2^, the GFR threshold that differentiates between stages 1 and 2 CKD. This difference by HIV serostatus was statistically significant among men 50–60 years of age, suggesting the possibility of earlier onset of reduced GFR in HIV(+) men. Only 23 men in the study had chronic hepatitis B virus (HBV) infection, a group too small to analyze statistically; in this group mean iGFR was lower for men with chronic HBV than for men without, both among HIV(−) men (means = 98 vs. 107 ml/min/1.73 m2; n = 4 and 254, respectively) and HIV(+) men (means = 98 vs. 109 ml/min/1.73 m2; n = 19 and 436, respectively).

### Factors Related to GFR

As shown in **Table 2**, in the total study population older age, HCV infection and history of AIDS were all significantly associated with lower mean iGFR. The effect of HCV infection was independent of HIV status (data not shown). The effect of age was estimated at −10.33 ml/min/1.73 m^2^ per decade. The effect of black race was of borderline significance (p = 0.09), with an effect similar to that of diabetes (although the latter was not significant in the multivariable analysis). Black race was significantly associated with iGFR ≤90 ml/min/1.73 m^2^ (**Table S1)**, as were the factors associated with low mean iGFR as above.

**Table 2:** Factors associated with mean iohexol-based GFR (iGFR) in the Multicenter AIDS Cohort Study, results from linear regression models.

		Univariate Analysis		Multivariate Analysis	
Characteristic				All	HIV-infected
		iGFR mean (S.E.)*	P-value	Diff (95% CI)*	Diff (95% CI)*
Age	<40 yr	123 (2.5)		ND	ND
	40–49 yr	113 (1.5)	**0.004**		
	50–59 yr	108 (1.4)	**<0.001**		
	≥60 yr	93 (2.0)	**<0.001**		
Age (per 10 years)		not applicable	**<0.001**	−**10.3 (−12.4,** −**8.3)**	−**10.8 (−13.8,** −**7.9)**
Race	Non-black	108 (1.1)		Ref	Ref
	Black	108 (1.6)	0.69	−3.5 (−7.4, 0.2)	−3.3 (−7.9, 1.4)
HIV and AIDS	No HIV	107 (1.4)		Ref	
	HIV(+) without AIDS	110 (1.2)	0.13	<0.1 (−3.4, 3.5)	Ref
	HIV(+) with AIDS	101 (3.4)	0.07	−**6.8 (−12.9,** −**0.7)**	−5.9 (−11.9, 0.2)
Hepatitis C virus	Not infected	109 (1.0)		Ref	Ref
	Infected	102 (2.6)	**0.006**	−**5.3 (−10.3,** −**0.4)**	−**6.4 (−12.3,** −**0.6)**
History of diabetes	No	109 (1.0)		Ref	Ref
	Yes	104 (2.5)	**0.042**	−2.1 (−6.6, 2.4)	−1.5 (−7.4, 4.4)
History of hypertension	No	111 (1.4)		Ref	Ref
	Yes	106 (1.2)	**0.008**	2.4 (−1.4, 6.2)	1.02 (−3.6, 5.6)
History of dyslipidemia	No	108 (3.3)		ND	ND
	Yes	108 (0.9)	0.97		
Proteinuria**	No	111 (0.9)		ND	ND
	Yes	87 (2.8)	**<0.001**		
HIV RNA	Undetectable	108 (1.3)		ND	Ref
	Detectable	111 (2.7)	0.37		−1.7 (−7.4, 3.9)
CD4 T cell count/uL	<300	111 (3.3)		ND	Ref
	300–500	106 (2.5)	0.12		−5.7 (−12.4, 1.1)
	>500	109 (1.4)	0.54		−4.1 (−10.5, 2.4)
HAART regimen	PI	108 (1.3)		ND	ND
	NNRTI but no PI	110 (2.6)	0.65		
HAART duration (per year)		not applicable	0.12	ND	−0.1 (−0.9, 0.5)
Tenofovir use	Never	110 (2.5)		ND	Ref
	Former	100 (3.9)	**0.010**		−**8.7 (−16.1,** −**1.3)**
	Current	110 (1.4)	0.85		−3.5 (−8.9, 1.8)
Tenofovir exposure	None	110 (2.5)		ND	ND
	>0 to 4 years	109 (1.8)	0.62		
	>4 years	107 (1.9)	0.32		

*Data given in ml/min/1.73 m^2^.

**Bold** indicates significant at P<.05.

ND = not included in the multivariate analysis.

Ref = reference value for the variable.

**Not included in multivariate analysis because of lack of independence from the other variables.

PI = protease inhibitor.

NNRTI = non-nucleoside reverse transcriptase inhibitor.

Among HIV(+) men, former tenofovir users also had significantly lower mean iGFRs than men who had never used this medication (**Table 2**). Mean iGFR did not differ significantly by current CD4 T-cell count, plasma HIV RNA level (detectable vs. undetectable), current tenofovir use (vs. never), duration of tenofovir use (none vs. 0–4 yr vs. >4 yr; univariate analysis only), HAART type (PI- vs NNRTI-based; univariate analysis only), or duration of HAART use. Compared to HIV(+) men with no history of AIDS, mean iGFR was lower among HIV(+) men with a prior AIDS diagnosis (difference = −5.91 ml/min/1.73 m^2^; p = .06). Proteinuria was also significantly associated with lower iGFR values (univariate analysis only). Among men with detectable plasma HIV RNA, there was no significant correlation between this measurement and iGFR (data not shown).

**Figure 2:**
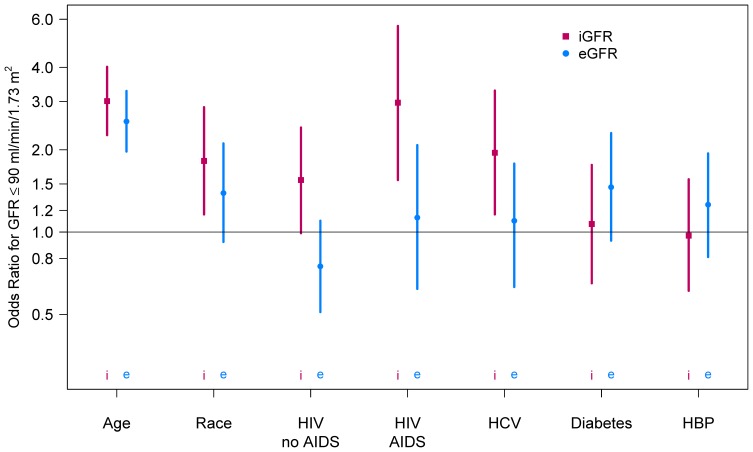
Comparative analysis of risk factors for low GFR (≤90 ml/min/1.73 m^2^) in MACS participants, using iohexol-based GFR (blue) or estimated GFR (brown). Odds ratios (solid boxes) and 95 percent confidence intervals (bars) were obtained from multivariate logistic regression models. HCV = Hepatitis C Virus infection. Diabetes = diabetes mellitus. HBP = high blood pressure.

Factors associated with iGFR ≤90 ml/min/1.73 m^2^ were the same as those associated with lower mean iGFR (i.e., age, HCV infection, and a history of AIDS) with the addition of black race and, of borderline significance, HIV infection without a history of AIDS (**Figure 2**, dark lines). In contrast, use of CKD-EPI eGFR failed to identify black race, AIDS history, and HCV infection as significant predictors of low eGFR (**Figure 2**, light lines). Low GFR by either method was not significantly associated with CD4 T-cell count, plasma HIV RNA (detectable vs. undetectable), duration of HAART use, or tenofovir use (**Table S1** and data not shown).

Using eGFR instead of iGFR resulted in differences in the percentage of men classified as having low GFR. While 21.8% and 19.0% of HIV(+) and HIV(−) men, respectively, had iGFR ≤90 ml/min/1.73 m^2^, 23.7% and 32.9%, respectively, had eGFR below this value. The greater difference between eGFR and iGFR values observed among HIV(−) men largely resulted from downward misclassification by eGFR: 18% of HIV(−) men had eGFR ≤90 and iGFR >90 ml/min/1.73 m^2^, but the reverse (i.e., upward misclassification by eGFR) was true for only 4% of HIV(−) men (p<0.01). This bias was not seen among HIV(+) men, for whom the corresponding rates of eGFR downward and upward misclassification were similar: 10% and 8%, respectively (p = 0.32). This differential misclassification affected the inferences about the relationship between HIV, age and low GFR. After adjusting for race and HCV infection, HIV(+) men aged 50–60 were significantly more likely to have a low iGFR than men younger than 50 years (OR = 2.44; 95% confidence interval (CI), 1.43–4.18), but this was not true for HIV(−) men of similar age (OR = 0.89, 95% CI, 0.34–2.33). These estimates were not affected by excluding men who had a history of AIDS.

### Prediction of Chronic Kidney Disease (CKD) using iGFR and eGFR

**Figure 3:**
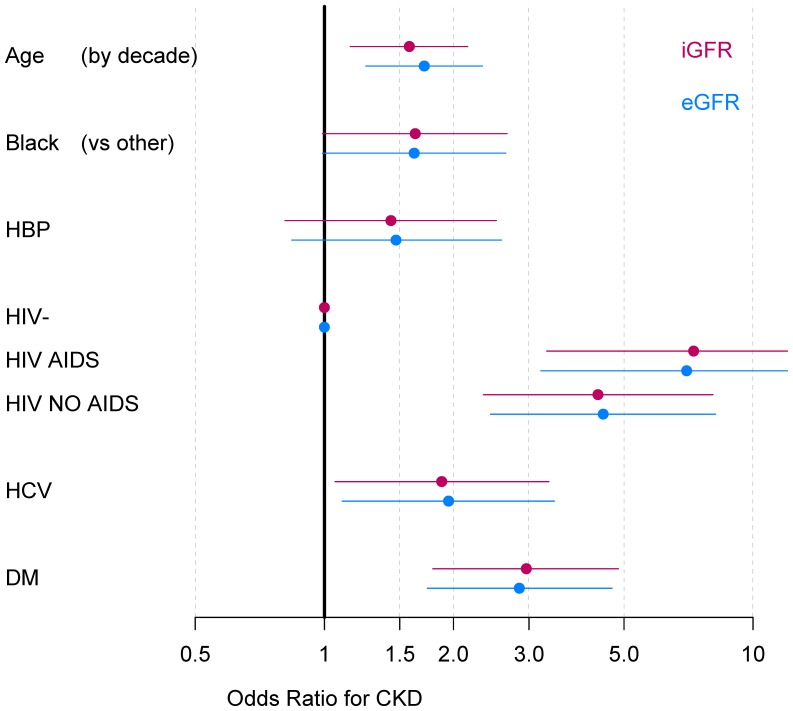
Multivariate predictions of CKD stage 1 or greater using either iohexol-based GFR (iGFR) or estimated GFR (eGFR), for (A) all participants and (B) HIV-infected (HIV(+)) participants only. Odds ratios (solid boxes) and 95 percent confidence intervals (bars) were obtained from multivariate logistic regression models.

There were 706 men who had both iGFR and eGFR (CKD-Epi) measurements available and thus could be classified according to the National Kidney Foundation stages of CKD using both measurements (**Table S2).** There were 104 men with at least stage 1 CKD by iGFR and 108 by eGFR; virtually all (598/602) men who did not have CKD using either method were concordantly classified. Approximately 6.2% of HIV(−) and 20.4% of HIV(+) men met criteria for CKD stage 1 or worse; about 2/3 of these men were concordantly classified, and classifications by eGFR and iGFR were very similar except for 4 men who had stage 3 by eGFR and no CKD by iGFR. As shown in **Figure 3**, odds ratios for factors associated with any CKD were virtually identical using iGFR and eGFR, except that current use of tenofovir was significant using iGFR and only borderline significant using eGFR.

## Discussion

Iohexol-based measurement of GFR (iGFR), though accepted as an accurate reference method, has been used primarily in populations with diminished kidney function [41], [42] or, in small studies, normal GFR [43], [44]. Recently, one study of 200 HIV(+) people with a range of plasma HIV RNA and GFR values found a good correlation between CKD-Epi eGFR and iGFR, providing some validation of the CKD-Epi equation in HIV(+) persons [13], and another study of 99 HIV(+) people with mostly normal GFRs found similar results [19]. However, neither of these studies assessed predictors of reduced GFR or of CKD. Thus, the present study is the first large, population-based study of iGFR in persons with largely normal kidney function, the first to compare iGFR in otherwise similar people with and without HIV infection, and the first to evaluate the impact upon iGFR of important chronic co-morbidities known to predispose to renal function decline in the general population, as well as HIV-specific factors. Consequently, findings from this study have implications for both HIV(−) and HIV(+) persons.

The main HIV-related factor associated with lower iGFR was a history of clinically-defined AIDS. HIV(+) men receiving HAART did not have significantly lower mean iGFR than HIV(−) men. However, compared to HIV(−) men, HIV(+) men had lower bottom quartiles of iGFR values by age, a higher proportion of iGFR values ≤90 or <60 ml/min/1.73 m^2^, and occurrence of iGFR ≤90 ml/min/1.73 m^2^ at a significantly younger age cross-sectionally. These HIV-related differences were partially explained by the effects of AIDS, chronic HCV infection (which was slightly more common in the HIV(+) group), and black race, but they persisted even after adjustment for these factors. Although former use of tenofovir was associated with lower iGFR, we found no consistent significant association between lower iGFR and current use or duration of use of tenofovir, a common association in eGFR-based analyses. This finding may reflect a prevalence or survival bias in that some men may have discontinued tenofovir use due to renal toxicity, while those who experienced no toxicity continued to use this medication. The association of past tenofovir use with lower iGFR is consistent with this interpretation, but we had insufficient data to fully investigate this possibility.

Across the entire study population, HCV infection was significantly associated with lower values of several measures of kidney function evaluated with iGFR, including mean iGFR, proportion with low iGFR, and presence of CKD. Chronic HCV infection, with or without HIV infection, has been associated with kidney insufficiency or failure [45], [46]. We oversampled HCV+ MACS participants, both HIV(+) and HIV(−), to evaluate this association. To our knowledge, this is the first study to demonstrate the effect of HCV on iGFR among people without prevalent CKD who had predominantly normal GFRs. This finding merits further investigation and could affect the optimal timing of intervention with definitive HCV therapy. Black race was significantly associated with lower iGFR in some of our analytic models.

Although both iGFR and eGFR were inversely associated with age, as expected [47], the associations of HCV infection, history of AIDS, and race with GFR ≤90 ml/min/1.73 m^2^ were observed only with iGFR. Thus, iGFR was more sensitive than eGFR in the identification of risk factors for mildly diminished GFR. These differences may reflect the finding that GFR ≤90 ml/min/1.73 m^2^ was approximately 25% less common using iGFR than the CKD-Epi eGFR. It is not clear why CKD-Epi-related eGFR misclassification about the 90 ml/min/1.73 m^2^ threshold was more preferentially downward in HIV(−) men than in HIV(+) men, since the overall eGFR distribution was about 7–10% lower than iGFR regardless of HIV serostatus, consistent with previous reports using inulin [48] isotopic methods [15], [16], [18], or iohexol [17], [44]. Further work in this area is forthcoming from our group. While these data suggest that iGFR may be more accurate than eGFR for identifying early CKD and its associated risk factors, further studies, particularly longitudinal studies, will be needed to confirm this interpretation. In this context, it is noteworthy that a recent report demonstrated that longitudinal iGFR measurement, but none of seven tested eGFR methods, accurately characterized GFR decline in a diabetic population [49]. On the other hand, in the present study estimates of CKD prevalence and predictors were quite similar using eGFR and iGFR. This finding provides some measure of validation supporting use of the CKD-Epi equation to assess CKD stage among HIV(+) people, particularly if proteinuria is also measured.

A limitation of the present study is that causality with low iGFR cannot be inferred from cross-sectional associations. Furthermore, our findings may not be generalizable to women and to persons with untreated HIV infection, although the majority of HIV(+) persons in developed countries now receive ART relatively soon after HIV diagnosis. Our definitions of chronic co-morbidities, which included men with both active and treated disease, may have limited our ability to ascertain the impact of these co-morbidities on GFR. Some men in our cohort who had kidney disease may not have participated in this study, which could have affected our estimation of factors associated with low GFR. Finally, iGFR measurement is too complex for routine clinical use. However, it provides valid and accurate measurements in the vast majority of cases, and is therefore well suited to population-based research such as the derivation of improved equations for estimating GFR in HIV(+) populations (Schwartz, G.J., et al, in preparation).

Despite these limitations, the important associations observed in this study would not have been seen if only eGFR measurements had been analyzed. In particular, we found a higher frequency of GFR ≤90 ml/min/1.73 m^2^ among HIV(+) persons receiving HAART compared to HIV(−)uninfected persons, especially in those who were older, or had HCV co-infection or a history of AIDS; these findings support close monitoring of kidney function in this population. The extent to which HIV infection and/or ART use may hasten these declines is not yet known. However, we found evidence of lower iGFRs among HIV(+) men aged 50–60 than HIV(−) men of this age, and earlier recognition of subtle impairments in GFR could have considerable clinical value. Longitudinal studies utilizing iGFR will be needed to optimize definitions of mild-to-moderate kidney impairment and to ascertain more fully the clinical implications of these definitions.

## Supporting Information

Table S1 **Multivariate analysis of factors associated with presence of iGFR ≤90 ml/min/1.73 m^2^ in study population.** NS = not significant. HCV = Hepatitis C Virus. ND = not included in the multivariate analysis.
(DOCX)

Table S2 **Classifications of CKD stage by eGFR and iGFR in total study population as well as HIV(−) and HIV(+) subpopulations.** *number in entire study population (number in HIV- men/number in HIV+ men).
(DOCX)
