# Supplementary material for: Factors Affecting Glomerular Filtration Rate, as Measured by Iohexol Disappearance, in Men with or at Risk for HIV Infection
Source: PLoS One. 2014 Feb 7;9(2):e86311. doi: 10.1371/journal.pone.0086311 (PMC3917840; doi:10.1371/journal.pone.0086311)
Supplement: Table S1 — Multivariate analysis of factors associated with presence of iGFR ≤90 ml/min/1.73 m2 in study population. NS = not significant. HCV = Hepatitis C Virus. ND = not included in the multivariate analysis. (DOCX) [file pone.0086311.s001.docx]

Table S1. Multivariate analysis of factors associated with presence of iGFR ≤90 ml/min/1.73 m^2^ in study population.

|  | **All Men** | | **HIV(+) Men** | |
| --- | --- | --- | --- | --- |
| **Effect** | **OR (95% CI)** | **P value** | **OR (95% CI)** | **P value** |
| Age (by decade) | 3.01 (2.26-4.00) | **<.001** | 2.36 (1.65-3.38) | **< .001** |
| Race (black vs others) | 1.82 (1.16-2.86) | **.010** | 2.07 (1.20-3.57) | **.009** |
| HIV(+) without history of AIDS (vs HIV(-)) | 1.55 (0.99-2.41) | .055 | ND |  |
| HIV(+) with history of AIDS (vs HIV(-)) | 2.97 (1.55-5.67) | **.001** | ND |  |
| HIV(+) with history of AIDS (vs HIV(+) with no history of AIDS) | ND |  | 1.88 (1.01-3.49) | **.047** |
| HCV (+ vs -) | 1.95 (1.16-3.29) | **.012** | 2.00 (1.09-3.67) | **.025** |
| History of diabetes (yes vs no) | 1.07 (0.65-1.76) | NS | 0.84 (0.46-1.54) | NS |
| History of hypertension (yes vs no) | 0.97 (0.61-1.56) | NS | 1.35 (0.76-2.41) | NS |
| CD4 T-cell count (300-500 vs <300/ul) | ND |  | 1.35 (0.61-2.96) | NS |
| CD4 T-cell count (>500 vs <300/ul) | ND |  | 1.10 (0.51-2.36) | NS |
| Plasma HIV RNA (detectable vs undetectable) | ND |  | 1.01 (0.51-1.99) | NS |
| Tenofovir use (former vs never) | ND |  | 1.20 (0.54-2.64) | NS |
| Tenofovir use (current vs never) | ND |  | 1.15 (0.62-2.12) | NS |
| HAART duration (per year) | ND |  | 1.06 (0.99-1.15) | NS |

NS= not significant

HCV= Hepatitis C Virus

ND= not included in the multivariate analysis
