# Supplementary material for: Factors Affecting Glomerular Filtration Rate, as Measured by Iohexol Disappearance, in Men with or at Risk for HIV Infection
Source: PLoS One. 2014 Feb 7;9(2):e86311. doi: 10.1371/journal.pone.0086311 (PMC3917840; doi:10.1371/journal.pone.0086311)
Supplement: Table S2 — Classifications of CKD stage by eGFR and iGFR in total study population as well as HIV(−) and HIV(+) subpopulations. *number in entire study population (number in HIV- men/number in HIV+ men). (DOCX) [file pone.0086311.s002.docx]

Table S2. Classifications of CKD stage by eGFR and iGFR in total study population as well as HIV(-) and HIV(+) subpopulations.

|  |  | CKD Stage by iGFR | | | | | Total |
| --- | --- | --- | --- | --- | --- | --- | --- |
|  |  | None | 1 | 2 | 3 | 4 |  |
| CKD Stage by eGFR | None | 598* | 0 | 0 | 0 | 0 | 598 |
|  |  | (240/358) |  |  |  |  | (240/358) |
|  | 1 | 0 | 37 | 12 | 0 | 0 | 49 |
|  |  |  | (7/30) | (0/12) |  |  | (7/42) |
|  | 2 | 0 | 13 | 13 | 4 | 0 | 30 |
|  |  |  | (0/13) | (1/12) | (3/1) |  | (4/26) |
|  | 3 | 4 | 0 | 7 | 17 | 0 | 28 |
|  |  | (1/3) |  | (0/7) | (3/14) |  | (4/24) |
|  | 4 | 0 | 0 | 0 | 1 | 0 | 1 (1/0) |
|  |  |  |  |  | (1/0) |  |  |
| Total |  | 602 | 50 | 32 | 22 | 0 | 706 |
|  |  | (241/361) | (7/43) | (1/31) | (7/15) |  | (256/450) |

*number in entire study population (number in HIV(-) men /number in HIV(+) men)
